# Patient choice in colorectal cancer treatment – A systematic review and narrative synthesis of attribute‐based stated preference studies

**DOI:** 10.1111/codi.16242

**Published:** 2022-07-18

**Authors:** Mikolaj Kowal, Francesca Douglas, David Jayne, David Meads

**Affiliations:** ^1^ The John Goligher Colorectal Surgery Unit St. James's University Hospital Leeds UK; ^2^ Leeds Institute of Medical Research, Faculty of Medicine and Health University of Leeds Leeds UK; ^3^ Leeds Institute for Health Sciences University of Leeds Leeds UK

**Keywords:** colonic neoplasms, colorectal neoplasms, humans, patient preference, research

## Abstract

**Aim:**

The global burden of colorectal cancer (CRC) is set to increase by 60% by 2030. An aging population and increasing treatment complexity add difficulties for patients and clinicians in CRC management. Patient preferences can be investigated using attribute‐based stated preference (AbSP) techniques to explore trade‐offs between different treatments. These techniques include discrete‐choice experiments (DCEs), conjoint analysis and time‐trade off (TTO) methods. This systematic review with a narrative synthesis aimed to determine the use and design of AbSP studies in CRC treatment and to identify patient choice themes.

**Methods:**

The searches were performed using MEDLINE, Embase, PsycInfo and Cochrane Library in March 2021. All manuscripts featuring the use of AbSP techniques in CRC treatment were included. Data synthesis was performed using a narrative approach.

**Results:**

The search strategy returned 271 articles. Eighteen AbSP studies were included featuring 1890 patients and 296 clinicians. AbSP techniques compromised DCE (38.9%, *n* = 7), TTO (38.9%, *n* = 7) and conjoint analysis (22.2%, *n* = 4). Eleven studies (61.1%) involved piloting of tasks and the average task completion rate was 75%. CRC treatments included chemotherapy (33%, *n* = 6), combined treatments (33%, *n* = 6), surgery (17%, *n* = 3), targeted therapy (11%, *n* = 2) and radiotherapy (6%, *n* = 1). The most examined domain was physical health, investigated with 49 (59.8%) attributes.

**Conclusions:**

Life expectancy was the main attribute in chemotherapy treatment. With surgery, patients were willing to trade life‐expectancy to avoid adverse outcomes or a permanent stoma. Communication skills, treatment cost, and clinicians' views were important attributes for patients in cancer services. Further research in the elderly population, and other quality of life domains, are needed to deliver patient‐centred CRC care.

## INTRODUCTION

Colorectal cancer (CRC) involves 11% of cancer diagnosis globally and is the third most common cancer in the world [1]. Novel treatment modalities, such as targeted therapies, in addition to the classic approaches of surgery, radiotherapy and chemotherapy, have added to the complexity of CRC management [2]. An aging population has resulted in almost half of CRC cases occurring in patients over 75 years, demanding careful patient selection for each treatment modality [3, 4]. With the global burden of CRC expected to increase by 60% by 2030, a clear understanding of patient choice is required to ensure that management is aligned to patients' expectations [5].

Patient‐centred care has been adopted as the preferred approach by healthcare systems and in the UK is a key high‐quality care indicator [6]. This model places the patient at the centre of the decision‐making process. In CRC management, patients are often required to make difficult choices. The choice they make is dependent upon their preferences and how they weigh‐up different aspects of treatment and trade‐off certain attributes. For example, an adult patient with moderate frailty might have to decide between a right hemicolectomy and potential cure, but with the risk of postoperative morbidity, against conservative management with inevitable cancer progression, but without immediate impact on quality of life.

To investigate preferences that patients have towards CRC treatments, attribute‐based stated preference (AbSP) techniques can be utilised to explore trade‐offs between different treatments. These techniques include discrete‐choice experiments (DCEs), conjoint analysis, best‐worst scaling (BWS) studies, and time‐trade off (TTO) methods. DCEs and conjoint analyses present respondents with a series of choices between two or more (treatment) options, each of which is described in terms of attributes (which may be outcomes and risks). The attributes are split into levels, often describing the severity of risk within healthcare research. Respondents weigh up the pros and cons of each option and choose which, on balance, they feel offers them the greatest value [7, 8]. The results are used to calculate the significance of each attribute and can be used for economical estimates for the willingness to pay for an attribute unit change. BWS studies establish respondents' relative preference for treatment and service attributes by asking them to rank and rate aspects or state which aspects are the worst and best [9]. The responses enable an exploration of how individuals value different characteristics of a service or treatment and if they are willing to trade between those characteristics. The TTO method focuses on establishing the degree to which respondents are willing to trade off quality and quantity of life. Individuals are presented with a series of choices between a period in full health and a longer period but in an imperfect health scenario. The burden attributed to the imperfect health scenario is determined by the amount of time they are willing to exchange for perfect health. TTOs establish the value of the imperfect health scenario and are the most common method for calculating quality of life weightings [10].

Current evidence for AbSP studies in CRC treatment is limited and there are no current reviews focusing on AbSP techniques in this field. This systematic review with a narrative synthesis aimed to identify the different AbSP techniques used to study CRC treatment, the main patient preference themes, and whether patients were willing to choose aggressive treatments, at the expense of short‐term morbidity, to improve life expectancy.

The aim of this study was to determine the current use and design of AbSP in CRC treatment and to identify the main themes of patient choices in chemotherapy, combination treatments, surgery, targeted therapies and radiotherapy. Our primary objective was to determine the current AbSP techniques used to investigate patient preference in CRC treatment and to assess the feasibility of preference elicitation in this study group evidenced by completion rates, missing data and assessments of validity.

The secondary aim was to determine the main themes of patient preference in CRC treatment.

## METHODS

### Study design and participants

The protocol for this systematic review was guided by the PRISMA and AMSTAR 2 guidelines and was prospectively registered with PROSPERO (registration no. CRD42021245077) [11, 12]. To provide the most comprehensive review of the literature in this field, we aimed to include all manuscripts meeting study type criteria which featured the use of AbSP techniques (DCE, TTO, BWS or conjoint analysis) in CRC treatment. All publication and study types were considered for inclusion to enrich the volume of applicable studies and reduce publication bias, including literature such as conference abstracts or editorials. Case series, cohort and case control studies and randomised control trials were included. Referenced studies within identified literature (specifically systematic reviews) were also considered for inclusion in the review by searching citations forwards and backwards. The participants were adult patients or clinicians involved in their care over the age of 18 that have been involved in an AbSP study as part of CRC treatment preferences. Exclusion criteria involved other types of preference studies (non‐AbSP) and studies not involving CRC treatment (for example CRC screening). AbSP studies were defined as any method that used quantitative data to explore preferences and exclusion of non‐AbSP studies ensured that narrative data synthesis could be performed in this study.

### Systematic literature search

Embase (Ovid), MEDLINE (Ovid), PsycInfo (Ovid) and Cochrane Library databases were systematically searched. Search strategies were designed with input from a senior information specialist within the affiliated research institution. The studies published from inception of databases until March 2021 were considered for inclusion. All identified studies were reviewed against the inclusion and exclusion criteria to assess eligibility. Referenced studies within identified literature were accessed and considered for inclusion.

The systematic search screening was performed by two independent investigations (MK and FD) using the databases described. All studies published up until 29 March 2021 inclusive were considered for eligibility. Studies identified were analysed for relevance to the systematic review prior to full inspection. Any discrepancies between the independent investigators were addressed by a third investigator (DM) until consensus was achieved. The search strategies used are displayed in full in Appendix S1.

### Primary and secondary outcomes

The primary outcome was the use of AbSP studies in CRC treatment. This was investigated through a focus on study design, with specific reference to study type, patient inclusion criteria, survey design, statistical design, sample size and analysis methods. The checklist created by the conjoint analysis task force of the International Society for Pharmacoeconomics and Outcomes Research (ISPOR) was used to standardise methodology assessment [13]. The ISPOR checklist is a 40‐item guideline reviewing general and specific aspects of AbSP studies such as attribute and level setting and preference elicitation. It is designed to highlight good research practises within the field of AbSP studies. The feasibility of preference elicitation in this study group was assessed as evidenced by completion rates, missing data and assessments of validity.

The secondary outcomes focused on the attributes identified collectively by all AbSP studies. These were extracted from each identified study and categorised according to chemotherapy, combination treatments, surgery, targeted therapies and radiotherapy.

### Data extraction

Two independent investigators (MK & FD) extracted data using a standardised data collection proforma, which included the following data fields:

1. Demographics: Patient inclusion criteria, average age, country of origin, study sample and CRC treatment offered.
2. Interventions: Type of AbSP technique utilised.
3. Study characteristics: AbSP design, preference elicitation, attributes and levels set, statistical analysis and adherence to ISPOR checklist.
4. Outcomes: Statistically significant patient preferences for CRC treatment identified by each study. Study completion rates, missing data and assessments of validity.

### Data synthesis

Due to study heterogeneity and the nature of research examined, a narrative synthesis approach was chosen to analyse a wide range of studies in a meaningful manner. We did not perform a meta‐analysis of effect estimates. The synthesis was conducted in line with the Guidance on the Conduct of Narrative Synthesis in Systematic Reviews from the Economic and Social Research Council [14]. Included studies were tabulated and grouped according to chemotherapy, combination treatments, surgery, targeted therapies and radiotherapy. Attributes investigated by individual studies were categorised using the World Health Organisation (WHO) domains of health‐related quality of life (HRQoL) [15]. The quality assessment was examined for each study using the ISPOR checklist. The evidence provided by the included literature was then synthesised to provide a structured narrative that was relevant to the research question. Due to study heterogeneity, the application of a standard scoring tool for risk of bias assessment was not possible.

## RESULTS

### Included and excluded studies

**FIGURE 1:**
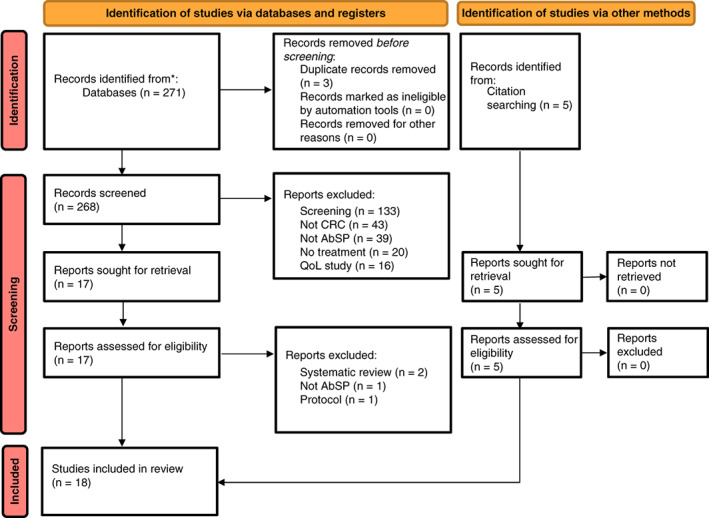
PRISMA flow diagram for study selection. Adapted from [11]

The search strategy returned 271 articles after duplication removal. Following abstract screening, 254 articles were excluded. The main reasons for exclusion were studies concerning screening for CRC, cancers other than colorectal, not involving an AbSP technique, or not featuring a treatment option. A further five studies meeting inclusion criteria were identified through reference searches within included systematic reviews. The 22 included studies subsequently underwent full review. Out of these, four studies were found to be systematic reviews or protocols for planned research and were therefore excluded from the analysis. This selection process is outlined in the PRISMA flowchart in Figure 1.

### Quality assessment of included studies

Quality assessment was performed using the ISPOR checklist for conjoint analysis applications in healthcare. For studies amenable to the checklist evaluation, the average number of met criteria was 33 (range 29–40) out of the total 40. The high level of agreement between included studies and the ISPOR checklist rated all studies as good quality. There were no studies deemed to be of lower quality, thus all studies were included in the analysis. The individual results for quality assessment and primary outcomes are shown in Table 1.

**TABLE 1:** The use of AbSP studies in CRC treatment grouped by AbSP type

Ref.	Study design	Study sample	Average age	Attributes	Tasks	Statistical analysis	Completion rate (%)	Validity tests	ISPOR score (/40)
Discrete choice experiments									
[23]	Pilot choice tasks	Stage 2 or 3 CRC patients *n* = 168	62	Survival, specific treatment side‐effects, cost of treatment, frequency of treatment administrations	Choice between two proposed treatments in a patient with stage 4 disease	Mixed logit model for preference weights and Wald test for statistical significance	43	N/A	31
[24]	Focus groups with patients and clinicians	New diagnosis of CRC *n* = 75	62	Continuity of care, understanding of diagnosis, treatment choice, time for therapy	Choice between two hypothetical cancer care services	Logistic regression model	53	Tested with additional control scenario	31
[29]	Literature review and qualitative interviews	CRC patients with at least one cycle of chemotherapy *n* = 108	60	Life expectancy, physical capacity, appearance, food digestion, waiting time	Choice between two proposed chemotherapy regimens	Logistic regression model	N/A	N/A	34
[22]	Pilot interviews with patients and clinicians	Metastatic CRC diagnosis *n* = 127	46	Progression‐free survival and specific treatment side‐effects	Choice between two proposed anti‐VEGF and anti‐EGFR treatments	Random‐parameters logit model	87	N/A	36
[19]	N/A	CRC with prior or planned chemotherapy *n* = 75	59	Likelihood and duration of chemotherapy side‐effects, cost per cycle of chemotherapy	Choice between two proposed chemotherapy regimens	Probit models	86	N/A	31
[30]	Qualitative interviews for main attribute selection	CRC patients *n* = 150	N/A	Life expectancy and chemotherapy specific side‐effects	Choice between two proposed chemotherapy regimens	Conditional logit model	N/A	N/A	N/A
[16]	Pilot survey to assess important aspects of decision‐making	CRC patient *n* = 107	66	Specialty training, communication, type of treating hospital and decision‐making style	Choice between two options of surgical centres offering surgery	Probit models	69	Test–retest reliability performed using a repeat survey	30
Conjoint analysis									
[31]	Based on prior study in breast cancer patients	CRC patients offered surgery or watch and wait strategy *n* = 94	62	Disease‐free survival, treatments with no colostomy, faecal and urinary incontinence, sexual dysfunction, concerns about cancer recurrence	Ranking task for most important aspects of care followed by choice between two surgical treatments	Hierarchical Bayes estimation was used to calculate the importance of each attribute	54	Subgroup analysis for attrition of certain patients	40
[18]	Values clarification method piloted in online survey	Rectal cancer patients eligible for radiotherapy and surgery *n* = 138	64	Survival, local recurrence, faecal incontinence, and male or female sexual dysfunction	Ranking task on how important they considered differences between best and worst probabilities of outcomes	Linear regression analyses	61	N/A	N/A
[32, 33]	N/A	Rectal cancer patients who had undergone LAR or APR *n* = 81	64	Survival, local control, sexual dysfunction and incontinence	Ranking task of 14 paired combinations of outcomes between surgery and radiotherapy	Pearson's r correlations, ANOVA and Kruskal–Wallis tests	86	Test and retest data compared using paired t‐tests	30
Time trade‐off									
[17]	Literature review and pilot trade‐off task	CRC patients receiving chemotherapy *n* = 118	61	Chemotherapy specific side‐effects, physical activity levels, mental health states, sleep and pain levels	Choice between two proposed chemotherapy regimens	Standard conditional logit form	97	N/A	35
[21]	Literature review for attributes	Rectal cancer patients following resection *n* = 47	59	Social interaction, fear of cancer recurrence, pain, fatigue, changes in bowel habits, and sexual dysfunction	Choice between two surgical treatment options	Nonparametric estimates of the survivor function	94	Not validated	29
[34]	Pilot interviews with patients	Rectal cancer patients following LAR or APR *n* = 120	68	Risk of incontinence, permanent stoma, daily faecal incontinence, monthly faecal incontinence, life expectancy	Trade‐off for life expectancy to avoid adjuvant therapy or APR	Trade‐off scores compared using Mann–Whitney U and Wilcoxon signed ranks tests	N/A	N/A	31
[20]	N/A	CRC (Dukes A–C) patients admitted for curative surgery *n* = 75	65	Risk of stoma and risk of adjuvant chemotherapy or radiotherapy	Trade‐off for life expectancy to avoid adjuvant therapy or stoma	McNemar tests or Wilcoxon signed rank tests	77	Test–retest reliability performed using a repeat survey	33
[35]	Clinicians from CRC MDT developed survey	CRC (Dukes A–D) patients who have undergone surgery *n* = 103	N/A	Bladder, bowel and sexual symptoms, faecal incontinence, a permanent colostomy, perianal soreness, pain in the pelvis, local cancer recurrence and frequent hospital visits	Trade‐off for life expectancy to avoid each adverse outcome of treatment	Wilcoxon's signed‐rank sum test	82	N/A	35
[36]	Pilot interviews with patients	CRC patients who had undergone surgery *n* = 100	N/A	Life expectancy, risk of stoma, risk of adjuvant therapy, risk of mortality from treatment	Trade‐off for life expectancy or gamble mortality to avoid stoma or adjuvant treatment	Wilcoxon's signed‐rank test.	91	N/A	29
[37]	Previously published study to elicit preferences	CRC stage 2 or 3 who had completed chemotherapy *n* = 123	65	Life expectancy	Trade‐off for life expectancy to make chemotherapy worthwhile	Linear regression	N/A	N/A	35

Abbreviations: AbSP, attribute‐based stated preference; CRC, colorectal cancer; ISPOR, International Society for Pharmacoeconomics and Outcomes Research; MDT, multidisciplinary team.

### Attribute‐based stated preference studies in colorectal cancer treatments

A total of 18 AbSP studies were identified in our search results with a total of 1890 patients with CRC and 296 clinicians involved in the treatment of CRC. The most frequently used techniques were DCE (38.9%, *n* = 7), TTO (38.9%, *n* = 7) and conjoint analysis (22.2%, *n* = 4). The included studies were published between 2003 and 2020 and a third had been published within the last 5 years (33.3%, *n* = 6).

### Analysis of methodology

Seven of the studies (38.9%) involved qualitative interviews conducted in preparation for a AbSP experiment. These included focus groups or interviews with patients or clinicians. Four studies (22.2%) included a method of piloting with surveys or pilot tasks to elicit main attributes for investigation. The remaining studies utilised expert advice or literature reviews to identify their attributes and levels for AbSP design. For example, Salkeld et al. [16]. utilised a pilot survey to assess the most important aspects of decision‐making from a pilot study group. The results were then selected and incorporated into a DCE task. A large degree of heterogeneity was observed in the inclusion criteria, with three studies recruiting patients with a past or present diagnosis of CRC (17%), two recruiting a specific stage of disease (11%), 10 recruiting patients with a completed or planned treatment (56%), and three requiring a specific stage and treatment for CRC (17%). No study focused on a specific age group apart from a general adult inclusion criterion. The average age of participants across all studies was 62 years.

**FIGURE 2:**
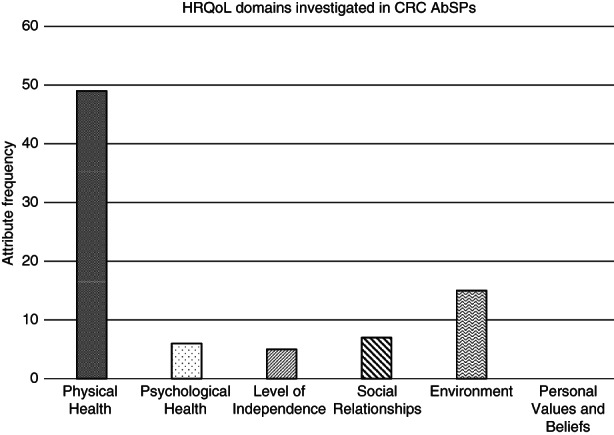
HRQoL domains investigated in CRC AbSPs. AbSPs, attribute‐based stated preference studies; CRC, colorectal cancer; HRQoL, Health‐related quality of life

The choice of attributes varied according to the treatment in question. The most frequently used attributes were specific treatment related side‐effects featuring in 11 studies (61%), followed by survival or life expectancy featuring in nine included papers (50%). Side‐effects mainly included nausea and vomiting for chemotherapy. Surgical treatments focused on faecal and urinary incontinence as well as sexual function. Three studies specifically asked for general health considerations such as physical activity and pain levels, out of which Osoba et al. was the only to enquire about mental health considerations [17]. Figure 2 shows the frequency of attribute investigation across all studies using the WHO domains of health‐related quality of life (HRQoL). The most examined domain was physical health, involving a total of 49 (59.8%) attributes. No studies included attributes related to personal values and beliefs.

All DCEs and conjoint analysis involved tasks which required a choice or ranking between two different treatments. AbSPs using trade‐off techniques all focused on the willingness to trade life expectancy for either making proposed treatment worthwhile or avoidance of adjuvant therapy or side‐effects.

### Feasibility of preference elicitation

We observed an average AbSP task completion rate of 75% (range 43%–97%) across all studies. Difficulties with data collection were uncommon, with one case by Pieterse et al. [18] of missing recording data due to logistical reasons. Validity was formally assessed and presented in five studies (28%) with the use of an additional control task. Out of these, three studies (17%) declared the use of test retest reliability calculated using paired *t*‐tests.

### Patient preferences in colorectal cancer treatment

The most investigated CRC treatments were chemotherapy and combinations of treatments in six studies (33%), with the latter involving a mixture of surgical and chemoradiation therapy. Three studies focused on surgery alone (17%), two investigated targeted therapy such as anti‐vascular endothelial growth factor (VEGF) agents (11%), and one examined the use of standalone radiotherapy (6%). The full results of patient preferences are displayed in Table 2.

**TABLE 2:** Patient preferences in CRC treatment

Ref.	Country	Treatment offered	Comparison	Main patient preferences	Main clinical preferences	Conclusions
Chemotherapy						
[24]	Italy	Chemotherapy counselling	Scenarios of different chemotherapy services	“Providing detailed information” and “high ability to understand” were most important attributes for chemotherapy counselling, followed by doctor's interpersonal skills	N/A	Results support a policy of strengthening medical doctors' capabilities to communicate with patients, providing them complete information and involving them in the clinical decisions
[29]	Germany	Chemotherapy	Scenarios of different chemotherapy regimes	Life expectancy was the most important attribute, “slightly changed appearance” and “no problems in food intake” were the most important adverse effects, patients with longer life expectancy valued quality of life effects more	N/A	Overall survival most important, patient preferences affected by cancer type, quality of life and surgery status
[19]	United States	Chemotherapy	Scenarios of chemotherapy with varied cost and side‐effect profile	Cost most important, followed by duration and severity of nausea and vomiting	N/A	Combined importance of avoiding nausea and vomiting outweighed the cost of therapy
[30]	United Kingdom	Chemotherapy	Scenarios of different chemotherapy regimes	Life expectancy was the most important attribute, patients preferred to trade‐off life expectancy for reduction in specific side‐effects	N/A	Negative values for pain, diarrhoea, fatigue, nausea and vomiting, and hair loss indicate that participants would not prefer a treatment that produces these toxicities and are willing to trade life expectancy
[17]	Canada	Chemotherapy	Scenarios of chemotherapy with varied side‐effect profile	Physical function most important, followed by social functioning, pain and fatigue	N/A	More precise interventions considering quality of life aspects are needed
[37]	Australia	Chemotherapy	Scenarios of chemotherapy with varied life expectancy	Small survival benefits judged important by most patients, 60% judged one month extra of survival important	N/A	Most subjects judged small survival benefits sufficient to make adjuvant chemotherapy worthwhile
Combination						
[32, 33]	Netherlands	Surgery and radiotherapy	Surgery vs. preoperative radiotherapy and surgery	Survival, local control, incontinence and sexual function most important to patients, most preferred surgery alone	Oncologists were willing to undergo preoperative radiotherapy for lower benefits than patients, also judged sexual function less important	Large difference in preference exist between patients and clinicians
[21]	Canada	Post‐op CRT	Surgery vs. surgery and postoperative CRT	Most patients willing to trade some risk of local recurrence for quality of life, 5% risk of local recurrence was the median switch point for preference	N/A	Substantial variation in patients’ preferences regarding postoperative CRT
[20]	Australia	Surgery and combinations of CRT	AR VS AR with postoperative radiotherapy, AR vs. AR with preoperative radiotherapy, AR vs. AR with chemotherapy, AR vs. AR with CRT, AR vs. APR	Strongest preference was to avoid a stoma, 65% willing to trade 34% of life expectancy to avoid this, patients were willing to trade most life expectancy to avoid chemoradiation	Colorectal surgeons' greatest preference was to avoid postoperative radiotherapy, oncologists less likely to trade life‐expectancy for avoidance of CRT	Results highlight the importance of determining patients' own preferences in the clinical encounter
[35]	Australia	Surgery and combinations of CRT with varied outcomes	AR vs. AR with postoperative radiotherapy, AR vs. AR with preoperative radiotherapy, AR vs. AR with chemotherapy, AR vs. AR with CRT, AR vs. APR	Majority of patients willing to trade life expectancy to avoid adverse treatment outcomes, the least accepted outcomes were for local recurrence in the pelvis followed closely by faecal incontinence	“Best fit” treatment for patients and surgeons was a LAR with postoperative chemotherapy, whereas for medical and radiation oncologists the best‐fit treatment was surgery alone	Local recurrence and faecal incontinence are considered the worst outcomes by patients and clinicians alike
[36]	Australia	Surgery and combinations of CRT	Open vs. laparoscopic surgery, APR vs. transanal excision, surgery vs. surgery with postoperative chemotherapy, APR vs. CRT	Majority of patients willing to trade life expectancy or gamble mortality for surgery alone to avoid chemotherapy or to avoid a permanent stoma,	Clinicians were more willing than patients to trade survival to avoid a permanent colostomy in favour of CRT	Unless patients' preferences are explicitly sought and incorporated into clinical decision‐making, patients may not receive the treatment that is best for them
Surgery						
[31]	Netherlands	Surgery	Watch and wait vs. surgery with permanent colostomy	Profile one preferred by patients (no surgery, less survival), difference of 2 years in disease‐free survival was considered least important	Patients ranked to have no surgery higher than clinicians	When confronted with different outcomes within one case description, patients find the duration of disease‐free survival the least important
[16]	Australia	Surgery	Scenarios of different surgical services and centres	Patients were very reluctant to trade off having a surgeon with additional training, treatment in a teaching hospital or having a surgeon who is easy to understand	N/A	Clinicians may have a better chance of meeting a patient's expectations about the process of care if they assess the patient's desire for knowledge
[34]	Netherlands	Surgery	APR vs. LAR	APR patients were less willing to give up life years to be without a permanent stoma than LAR patients, APR patients also preferred LAR above APR, but only if it involved a small risk of incontinence	N/A	Most patients preferred LAR above APR, even if LAR involved a risk of faecal incontinence
Targeted therapy						
[23]	Singapore	Targeted therapy	Anti‐VEGF vs. anti‐EGFR agents	Cost was most important to patients, followed by bleeding because of therapy, acne and survival	N/A	Patients were willing to trade off some degree of efficacy to avoid certain severity of side effects
[22]	United States	Targeted therapy	Anti‐VEGF vs. anti‐EGFR agents	Increasing survival was most important attribute, older patients placed greater weight on improving survival and avoiding gastrointestinal perforation and skin rash than did younger patients	On average, physicians were willing to tolerate higher risks than patients	Initiating or enhancing discussions about patient tolerance for toxicities, such as skin rash and gastrointestinal perforations, may help prescribe treatments that entail more appropriate benefit–risk trade‐offs
Radiotherapy						
[18]	Netherlands	Radiotherapy	Short course preoperative radiotherapy	Patients in the values clarification method group experienced lower regret and less impact of treatment harms at 6 months follow‐up	N/A	Being explicitly invited to think about treatment benefits and harms seems to help patients to live with treatment consequence

Abbreviations: APR, abdominoperineal resection; AR, anterior resection; CRT, chemoradiotherapy; EGFR, epidermal growth factor receptor; LAR, low anterior resection; VEGF, antivascular endothelial growth factor.

In chemotherapy treatments, life expectancy was the most important attribute when choosing treatments in most studies (4 out of 6 studies). Other important attributes included specific side‐effects, such as nausea and vomiting, and adverse outcomes on aspects such as physical capacity and personal appearance. Cost of treatment was an attribute included in the United States based study by Miller et al. and was found to be the most important factor in the decision process [19].

With regards to combination treatments, a mixture of treatment strategies was investigated including neoadjuvant and adjuvant chemoradiation, anterior resection, abdominoperineal resection, and local transanal excision. In four out of the six studies, patients were willing to trade life expectancy or gamble survival to avoid adjuvant therapy or a permanent stoma. More specifically, Harrison et al. found that 65% of patients were willing to trade 34% of life expectancy to avoid a stoma and Couture et al. concluded that a 5% risk of local recurrence was the switch point for decisions on adjuvant chemoradiation [20, 21]. Other adverse outcomes that were important to patients included incontinence and sexual function.

In targeted therapies, the literature compares anti‐VEGF and antiepidermal growth factor receptor for metastatic CRC. Gonzalez et al. found that increasing survival was the most important attribute, contrary to the results by Wong et al. where patients in Singapore placed most weight on the cost of treatment and the side‐effects profile [22, 23]. Older patients were also found to prioritise improving survival and avoiding side‐effects when compared to younger patients in a subgroup analysis.

Six of the included studies involved a comparison between patient and clinician views on the same preferences for CRC treatments (33%). This involved placing the clinicians in the situation of their patients and making choices within a theoretical scenario. All studies identified a large degree of variation between the two cohorts. Clinicians were more likely to undergo treatments with higher risks and were less likely to trade life expectancy to avoid adjuvant treatments or specific side‐effects.

Salkeld et al. and Rosato et al. focused their research on evaluating cancer services rather than specific treatment options [16, 24]. The former analysed surgical services with respect to surgeon experience, communication skills, and nature of surgical centres. The latter looked at chemotherapy services with emphasis on continuity of care, information provided and time to therapy. Both patient cohorts ranked communication skills as one of the most important attributes, with surgeon experience an important factor in surgical counselling, and quality of information provided important in chemotherapy counselling.

## DISCUSSION

To our knowledge, this is the first systematic review with a narrative synthesis of AbSP techniques for patient preferences in CRC treatment. We have identified the common techniques used, consisting of DCE, TTOs and conjoint analysis, which currently have moderate adherence to internationally recognised reporting checklists. Piloting of tasks is a common method in the design process. Initial developments, testing and optimisation are essential stages in the design of AbSP experiments which impact on the validity of the results [25, 26]. Our findings show that just over 50% of the studies include an aspect of this process, thus the methodology of AbSP experiments within the field of CRC could be improved. The main attributes chosen for CRC treatment have been identified, which typically involve side‐effects and life expectancy, focusing on the physical health domain of HRQoL. Preference elicitation is feasible and has a high average completion rate in the relatively young average age group presented in the current literature. Inclusion criteria vary from a past diagnosis of CRC to defined disease stages with specific treatment eligibility, with limited focus on age groups.

This systematic review with a narrative synthesis shows that the current literature investigating patient preferences in CRC focuses on chemotherapy and combination treatments. In chemotherapy, life expectancy is the most important aspect for patients. When surgery is offered, patients are often willing to trade off life expectancy to avoid a stoma or adjuvant therapies. They also place greater weight on outcomes such as incontinence and sexual function. In settings where cost is taken into consideration, patients always value the cost as the most important attribute. Furthermore, clinician's views vary when compared to patient choices, with clinicians being more likely to take risks and less likely to trade life expectancy. For cancer service evaluation, patients value communication skills as one of the most important attributes.

Our patient preference findings support the current literature from 2014 and 2015. A systematic review by Damm et al. included all studies concerning patient preferences for CRC treatment. The authors comment on large heterogeneity in patient preferences, specifically when examining personal factors such as age and gender. We have only included one AbSP study which focused on different age groups; thus we identify this as an area for further research in the field [27]. A further systematic review by Currie et al. [28]. identified eight studies concerning patient preferences in CRC. We support their conclusions in patients' willingness to trade life expectancy for adjuvant therapy and stoma avoidance. In addition to this, we identify the disparity with clinician's views, which is likely to impact on patient decision‐making and their satisfaction with outcomes and personal choices.

With regards to future research, we have identified a need for further analysis of patient preferences related to surgical interventions, with specific reference to elderly patients (age over 65). The average age across all included studies was 62 and no study focused on an inclusion criterion of age or frailty index score to capture this patient group. In addition to this, we recommend future research to involve a comprehensive assessment of HRQoL. Specifically, future studies should include attributes encompassing all HRQoL domains, as we have shown limited representation of domains such as psychological health and level of independence in the current literature. Research into all domains of life will enable better patient‐centred care. One third of the studies were published in the last 5 years, meaning that the majority of the literature does not reflect advances within CRC management. The current AbSPs do not investigate clinical scenarios involving new neoadjuvant or watch and wait treatment strategies. New advances in CRC treatment should be followed by AbSP studies to aid decision‐making for patients and clinicians.

The strengths of this systematic review include the use of a narrative synthesis to undertake a comprehensive review of all CRC treatments within a heterogenous population and study design. We have identified the main patient preferences across different CRC treatments, and have highlighted areas for future research. Our systematic review with narrative synthesis is novel in focusing on AbSP techniques and provides an update to the current literature, with numerous studies published since the last systematic review in the field. The limitations of the study are also recognised. The study heterogeneity prevented us from performing a meta‐analysis, although the provision of evidence‐based guidelines was not our aim. In particular, variation was observed within the stage of disease included in each study, potentially affecting the results of our narrative synthesis. However, our ability to account for this was limited as only two studies featured an inclusion criterion for disease stage. We also did not include studies concerning quality of life questionnaires or CRC screening, which may have added to the AbSP methodology and feasibility of preference elicitation analysis. The literature identified is limited in its origins to northern Europe, US, Canada, Australia and Singapore, which impacts on the generalisability of the results to a global level. Finally, we were unable to apply a formal risk of bias tool as we pragmatically assessed this utilising the ISPOR checklist.

## CONCLUSIONS

The current literature relating to patient preferences for CRC treatment involves DCE, conjoint analysis and trade‐off techniques. We have identified life expectancy as an important factor in chemotherapy regimens. In surgical treatments, patients are willing to trade life‐expectancy to avoid adverse outcomes or a permanent stoma. Further research in elderly patients is needed to assess their preferences when faced with a CRC diagnosis. Communication skills, cost of treatment, and clinician's' views are important attributes for patients and should be considered when developing cancer services.

## FUNDING INFORMATION

The systematic review described in this study received no funding.

## AUTHOR CONTRIBUTIONS

Study conception and design: MK, DJ, DM Data acquisition: MK, FD DraftingofManuscript:MK, FD, DJ, DM. Dataanalysisandinterpretation,criticalrevision, and final approval of the manuscript: all authors.

## ETHICAL APPROVAL

No ethical apporval was required for this study.

## CONFLICT OF INTEREST

None declared.

## Supporting information


Appendix S1

## Data Availability

Data sharing is not applicable to this article as no new data were created or analyzed in this study.
