# Supplementary material for: Patient choice in colorectal cancer treatment – A systematic review and narrative synthesis of attribute‐based stated preference studies
Source: Colorectal Dis. 2022 Jul 18;24(11):1295–307. doi: 10.1111/codi.16242 (PMC9796068; doi:10.1111/codi.16242)
Supplement: Supplementary file 1 — Appendix S1 [file CODI-24-1295-s001.docx]

**Appendix**

Supplementary 1: Systematic Review Search Strategy for OVID and Cochrane Library

| Search number | Keyword Search Strategy |
| --- | --- |
| 1 | colorectal cancer*.mp |
| 2 | colorectal neoplasm*.mp |
| 3 | colorectal tumo?r.mp |
| 4 | colorectal carcinoma .mp |
| 5 | colonic cancer*.mp |
| 6 | colonic neoplasm*.mp |
| 7 | colon neoplasm*.mp |
| 8 | rectal neoplasm*.mp |
| 9 | rectal cancer*.mp |
| 10 | colitis-associated neoplasm*.mp |
| 11 | sigmoid neoplasm*.mp |
| 12 | 1 OR 2 OR 3 OR 4 OR 5 OR 6 OR 7 OR 8 OR 9 OR 10 OR 11 |
| 13 | discrete choice*.mp |
| 14 | stated preference*.mp |
| 15 | choice behavio?r.mp |
| 16 | conjoint analysis.mp |
| 17 | (BWS or best-worst) .mp |
| 18 | (maximum difference or maxdiff or max-diff) .mp |
| 19 | (choice adj (based or model* or experiment* or behavio?r*)).mp |
| 20 | preference*.mp |
| 21 | 13 OR 14 OR 15 OR 16 OR 17 OR 18 OR 19 OR 20 |
| 22 | 21 AND 22 |

Cochrane Library Search Strategy

(colorectal cancer* OR colorectal neoplasm*

OR colorectal tumo?r OR colorectal carcinoma OR colonic cancer* OR colonic neoplasm* OR colon neoplasm* OR rectal neoplasm* OR rectal cancer* OR colitis-associated neoplasm* OR sigmoid neoplasm*) AND (discrete choice* OR stated preference* OR choice behavio?r OR conjoint analysis OR (BWS or best-worst) OR (maximum difference or maxdiff or max-diff) OR (choice adj (based or model* or experiment* or behavio?r*)) OR preference*)
